# Unlocking the Potential of Perillaldehyde: A Novel Mechanism for Chronic Myeloid Leukemia by Targeting HSP70

**DOI:** 10.3390/molecules30112294

**Published:** 2025-05-23

**Authors:** Miaomiao Zhang, Jinfeng Wang, Rongsong Jiang, Ming Liu, Weiyi Zhang

**Affiliations:** 1 School of Pharmacy, Xinjiang Medical University, Urumchi 830017, China; zmm@xjmu.edu.cn (M.Z.); wjf@xjmu.edu.cn (J.W.); jrs@stu.xjmu.edu.cn (R.J.); lmouc@ouc.edu.cn (M.L.); 2 Key Laboratory of Marine Drugs, Chinese Ministry of Education, School of Medicine and Pharmacy, Ocean University of China, Qingdao 266003, China; 3 Xinjiang Key Laboratory of Natural Medicines Active Components and Drug Release Technology, Urumchi 830017, China; 4 Xinjiang Key Laboratory of Biopharmaceuticals and Medical Devices, Urumchi 830017, China; 5 Engineering Research Center of Xinjiang and Central Asian Medicine Resources, Ministry of Education, Urumchi 830017, China

**Keywords:** perillaldehyde, chronic myeloid leukemia, apoptosis, autophagy, HSP70

## Abstract

Leukemia is a malignant tumor of the hematopoietic system. Approximately 15% of adult leukemias are chronic myeloid leukemias (CMLs), and this incidence increases annually. The BCR-ABL oncoprotein drives the initiation, promotion, and progression of CML. Although tyrosine kinase inhibitors (TKIs) are first-line therapies for CML, BCR-ABL-mediated drug resistance limits their clinical efficacy and patient prognosis. Perillaldehyde (PAE), a monoterpene and primary volatile oil from perilla, is a promising small-molecule candidate for degrading BCR-ABL and has potential medical applications. The molecular mechanism showed that PAE regulated the expression of autophagy- and apoptosis-related proteins in K562 cells. Confocal laser observation showed that PAE damaged the mitochondrial membrane potential and induced ROS generation. Further evaluations indicated that PAE targeted HSP70 and inactivated the phosphorylation of BCR-ABL, thereby inhibiting its downstream proteins. This study may produce a lead compound for CML therapy as PAE may be an effective treatment for further exploration.

## 1. Introduction

Chronic myeloid leukemia (CML) is a rare myeloproliferative tumor with an annual incidence of 1.0–1.5/10^5^ [1]. This lethal hematological malignancy originates from myeloid progenitor cells and is characterized by uncontrolled proliferation, multiple clinical manifestations, and aberrant hematopoietic progenitor cell proliferation [2]. It is well-known that CML can be treated with various drugs, including combination chemotherapy, which is commonly used. However, these treatments have side effects, and myelosuppression and infection-related toxic effects increase with prolonged treatment [3]. Since the development of BCR-ABL tyrosine kinase inhibitors (TKIs), CML has been treated effectively, and many patients experience long-term remission and live near-normal lives. Moreover, the 10-year survival rate has increased from approximately 20% to 80–90% [4]. Despite progress over the past two decades, 5–10% of patients treated with TKIs fail for resistance-related reasons [5]. TKI resistance is caused by multiple molecular mechanisms, including changes in the molecular drug target itself, changes in the tumor microenvironment, and immune cell dysfunction [6]. To avoid the serious side effects of chemotherapy and increase survival rates, researchers have focused on developing new cancer therapies and anticancer drugs for CML and improving the prognosis.

Natural products, characterized by their low toxicity and multi-target mechanisms of action, have demonstrated significant potential in cancer therapy, thereby offering novel insights and strategies for the treatment of cancer [7]. Perillaldehyde (PAE) is one of the most important essential oils extracted from the perilla plant (Perilla frutescens) [8]. Studies have demonstrated that PAE possesses antioxidant activity [9] and modulates the intracellular redox balance to mitigate oxidative stress-related damage [10]. Additionally, PAE has been found to protect against prostate cancer [11] and has anti-depressant [12] and anti-inflammatory effects [13]. Perilla frutescens leaves are commonly used as flavoring agents in stews, soups, and roasts [14]. They serve as raw materials for the production of essential oils and perfumes [15]. Although the existing literature has documented the ability of PAE to induce ferroptosis in acute myeloid leukemia (AML) cells [16], its efficacy and mechanisms in CML remain to be elucidated.

In recent years, heat shock protein 70 (HSP70), as an important molecular chaperone, has garnered extensive attention due to its pivotal role in cellular stress responses and tumor biology [17]. Under normal physiological conditions, HSP70 is involved in protein folding, translocation, and degradation, thereby maintaining intracellular protein homeostasis [18]. However, in tumor cells, the expression of HSP70 is often significantly upregulated, and it promotes cancer cell survival, proliferation, and drug resistance through various mechanisms [19]. Additionally, HSP70 participates in the regulation of the stability of cyclins, facilitating cell cycle progression and further driving cancer cell proliferation [20]. Therefore, HSP70 is an important tumor biomarker and a potential therapeutic target.

The K562 cell line, which harbors the BCR-ABL fusion gene responsible for uncontrolled cell proliferation, has been widely utilized in leukemia research [21]. This study demonstrates that perillaldehyde (PAE) exhibits cytotoxic effects on various cancer cells, particularly K562 cells, by significantly inhibiting cell proliferation, arresting the cell cycle, and inducing apoptosis and autophagy in K562 cells. Molecular mechanism studies suggest that PAE may exert its anti-tumor effects via the mitochondrial pathway, with HSP70 emerging as a potential target. Therefore, PAE may be a promising therapeutic approach. This study provides crucial theoretical and experimental foundations for the development of novel, low-toxicity, and highly effective anti-CML drugs.

## 2. Results

### 2.1. PAE Inhibited the Growth of K562 Cells In Vitro

**Figure 1:**
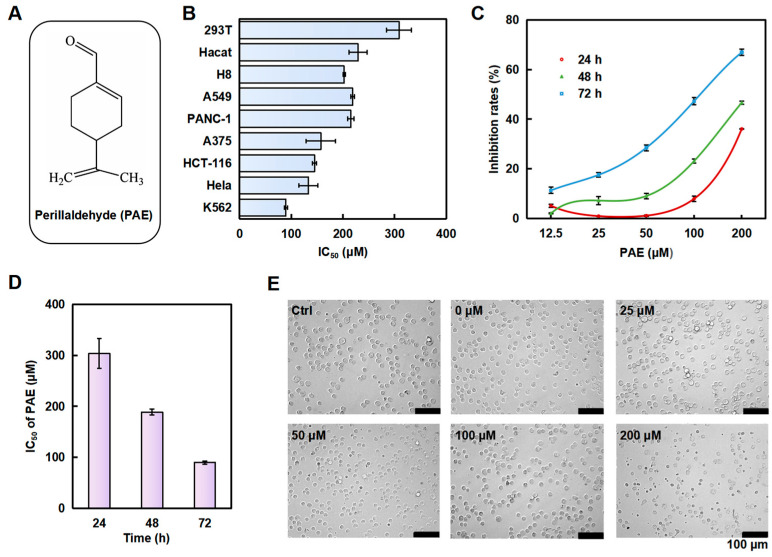
PAE inhibits the proliferation of K562 cells. (**A**) Structure of PAE. (**B**) Cells were treated with PAE for 72 h, and the cytotoxicity of PAE was evaluated (n = 3). (**C**,**D**) K562 cells were treated with PAE (0–200 µM, 24–72 h), then cell viability and IC50 values were calculated (n = 3). (**E**) Pictures showing the changes in cell morphology after PAE treatment for 24 h.

The chemical structure of PAE is presented in Figure 1A. In order to assay the specificity of PAE against different types of cells, tumor cells (A549, PANC-1, A375, HCT-116, Hela, and K562) and normal cells (293T, Hacat, and H8) were tested. As presented in Figure 1B, PAE had a better inhibitory effect on the human leukemia cell line K562 and exhibited less toxicity to normal cells. Moreover, PAE inhibited the proliferation of K562 cells (Figure 1C), and the 50% growth inhibition concentration (IC50) values of PAE against the K562 cell line were 303.3, 188.7, and 89.7 μM at 24, 48, and 72 h, respectively (Figure 1D). Treatment with PAE changed the morphological properties of K562 and decreased their population (Figure 1E). These results demonstrated that PAE could inhibit K562 cell growth in vitro.

### 2.2. PAE Induced G0/G1 Phase Arrest and Caspase-Dependent Apoptosis in K562 Cells

**Figure 2:**
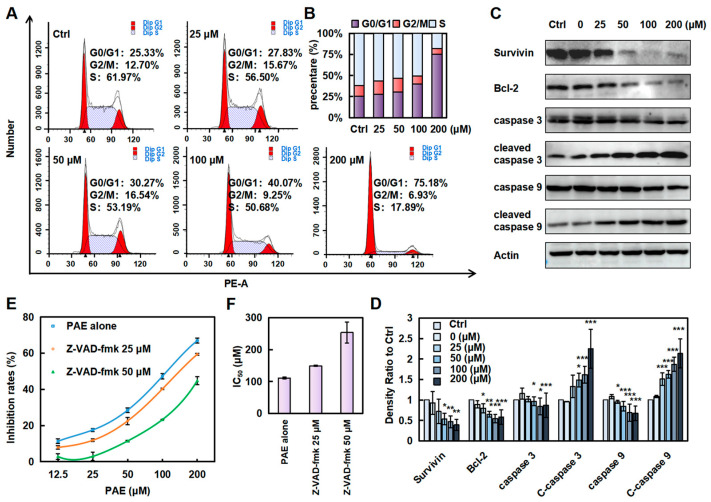
PAE arrested the cell cycle and induced apoptosis in K562 cells. (**A**,**B**) Cell cycle distribution analyzed by DNA content after PAE treatment (0–200 µM, 24 h, flow cytometry). (**C**,**D**) Apoptosis-related protein expression detected by Western blotting following PAE exposure (0–200 µM, 24 h). (**E**,**F**) Cell viability assessed with Z-VAD-fmk pretreatment (2 h) followed by PAE treatment (24 h). * *p* < 0.05, ** *p* < 0.01, *** *p* < 0.001 vs. Ctrl (n = 3).

To test whether cell cycle arrest contributed to PAE-induced proliferation inhibition, we further analyzed the cell cycle distribution by PI staining. Moreover, we found that PAE induced G0/G1 phase arrest in K562 cells in a concentration-dependent manner (Figure 2A,B). To further detect whether PAE induced apoptosis in K562 cells, we examined several apoptosis-related proteins. The results showed that the anti-apoptotic proteins survivin and Bcl-2 were decreased (Figure 2C,D and Figure S1).

In addition, the levels of cleaved caspase-3 and cleaved caspase-9 were significantly upregulated, while their proenzyme forms (caspase-3 and caspase-9) showed corresponding downregulation, collectively indicating the activation of apoptotic pathways in the experimental system. Meanwhile, pretreatment with the pan-caspase inhibitor Z-VAD-fmk significantly attenuated PAE-induced cytotoxicity in K562 cells, as evidenced by a marked increase in IC50 values from 111.0 μM to 253.7 μM (Figure 2E,F). This result suggested that caspase activation is an essential mechanism mediating PAE-triggered apoptotic cell death. Our findings collectively demonstrate that PAE induces caspase-dependent apoptosis in K562 cells.

### 2.3. PAE Induced Autophagy in K562 Cells

**Figure 3:**
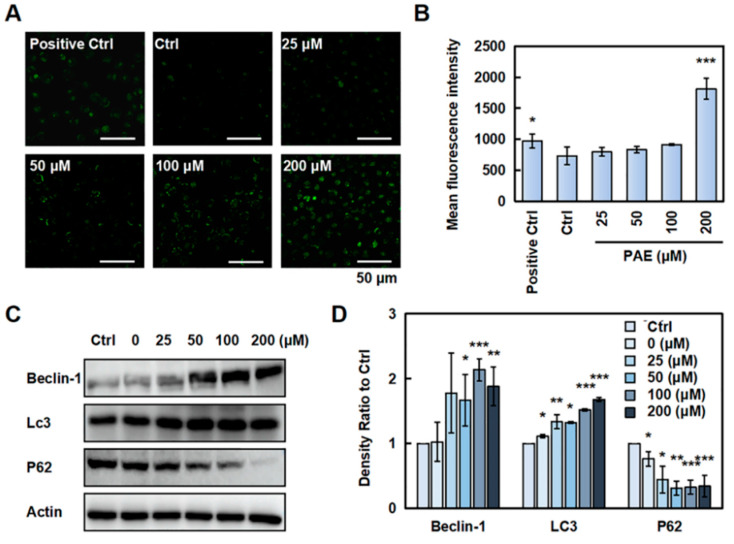
PAE-induced autophagy in K562 cells. (**A**,**B**) Cells were treated with PAE (0–200 µM) for 24 h, and a monodansylcadaverine (MDC) assay was used to detect the effect of PAE on autophagy in K562 cells (MDC, an eosinophilic dye, can specifically bind to autophagosomes to form green fluorescence). Histograms show the mean fluorescence intensity. (**C**,**D**) Cells were treated with 0–200 µM PAE for 24 h, and autophagy-related proteins were detected. * *p* < 0.05, ** *p* < 0.01, *** *p* < 0.001 vs. Ctrl (n = 3).

We first used confocal laser microscopy to detect whether PAE induced autophagy in K562 cells. As displayed in Figure 3A,B, green fluorescence was increased, suggesting that PAE could disrupt the induced autophagy in K562 cells. We further examined several autophagy-related proteins, and the results showed that Beclin-1 and Lc3 were increased. Meanwhile, P62 was decreased in the presence of PAE (Figure 3C,D and Figure S2). These results indicated that PAE induced autophagy in K562 cells.

### 2.4. PAE Disrupted Mitochondrial Function in K562 Cells

**Figure 4:**
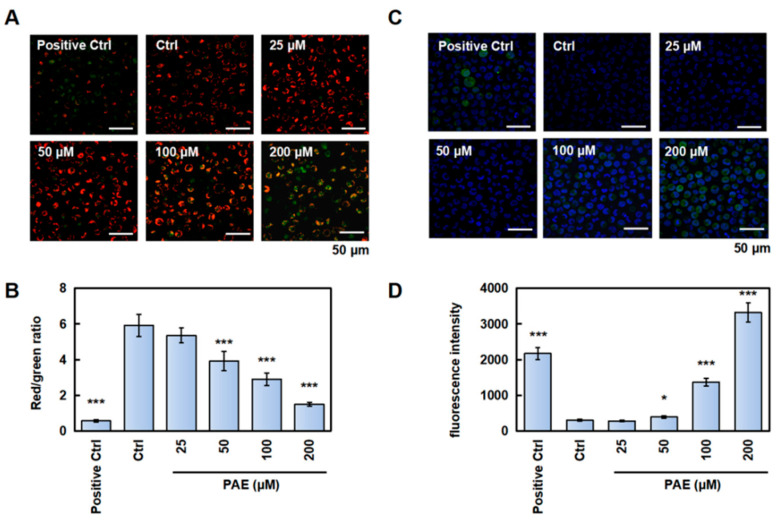
PAE disrupted mitochondrial function in K562 cells. (**A**,**B**) Cells were treated with PAE (0–200 µM, 24 h), stained with a JC-1 probe, and analyzed using laser scanning confocal microscopy (the green fluorescence represents the JC-1 monomer, and the red fluorescence represents the JC-1-aggregates). Histograms show the red/green ratio. (**C**,**D**) Cells were treated with PAE (0–200 µM, 24 h), and ROS levels were detected using laser scanning confocal microscopy (the green fluorescence represents the DCFH-DA, and the blue fluorescence represents the Hoechst 33342). Histograms show the mean fluorescence intensity. * *p* < 0.05, *** *p* < 0.001 vs. Ctrl (n = 3).

The mitochondrial membrane potential (MMP), a critical bioenergetic parameter reflecting the functional status of mitochondria, serves as both a key biomarker for cellular vitality and a central regulator of apoptotic cascades. Therefore, we tested the effect of PAE on the MMP using a JC-1 probe. As depicted in Figure 4A,B, when cells were exposed to PAE, the red/green ratio decreased, suggesting that PAE disrupted the MMP of K562 cells. ROS is closely related to cell apoptosis; therefore, we examined whether PAE stimulated ROS generation. After PAE exposure, the green fluorescence intensity increased in K562 cells (Figure 4C,D), indicating a PAE-induced increase in ROS generation. These results indicated that PAE disrupted the MMP and increased ROS levels in K562 cells.

### 2.5. PAE Downregulated the Phosphorylated BCR-ABL Protein and Inhibited Its Downstream Proteins in K562 Cells

**Figure 5:**
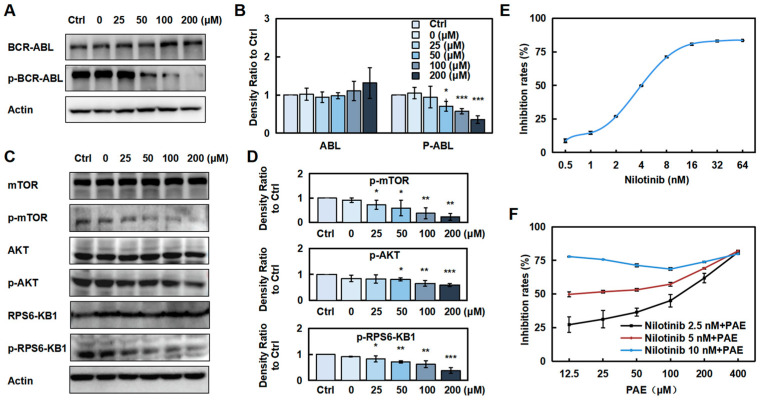
Prediction of the targets of 6-PAE. (**A**–**D**) Cells were treated with PAE, BCR-ABL, and pBCR-ABL, and the related proteins were examined. Histograms show the intensity of protein bands. (**E**,**F**) Cells were treated with nilotinib alone or in combination with PAE, and the IC50 and inhibition rates were detected. * *p* < 0.05, ** *p* < 0.01, *** *p* < 0.001 vs. Ctrl (n = 3).

Considering that BCR-ABL is a major pathogenic factor of CML, we investigated whether PAE affects its expression. As expected, a dose-dependent reduction in p-BCR-ABL protein levels in K562 cells was observed after PAE treatment (Figure 5A,B and Figure S3). To further elucidate the related downstream proteins regulated by BCR-ABL, we detected the AKT/mTOR/S6K1 pathway, which is also an important intracellular signaling pathway in tumorigenesis. The results showed that the phosphorylation of AKT, mTOR, and S6K1 was decreased (Figure 5C,D and Figure S4). These results indicate that PAE decreased the expression of the phosphorylated BCR-ABL protein and its downstream proteins in K562 cells.

Nilotinib, a BCR-ABL tyrosine kinase inhibitor, demonstrates potent anti-tumor efficacy. However, its clinical utility is constrained by dose-limiting toxicities, including cardiovascular complications and hematological abnormalities. Therefore, we aim to investigate whether PAE can mitigate the toxic effects of nilotinib. A combination of nilotinib and PAE was used to test whether PAE alone or in combination with nilotinib promoted K562 cell proliferation (the detected concentrations were close to its IC50, Figure 5E). The result showed that PAE acted synergistically to improve the inhibitory effect of nilotinib (Figure 5F).

### 2.6. DARTS Assay Reveals That PAE Targets HSP70

**Figure 6:**
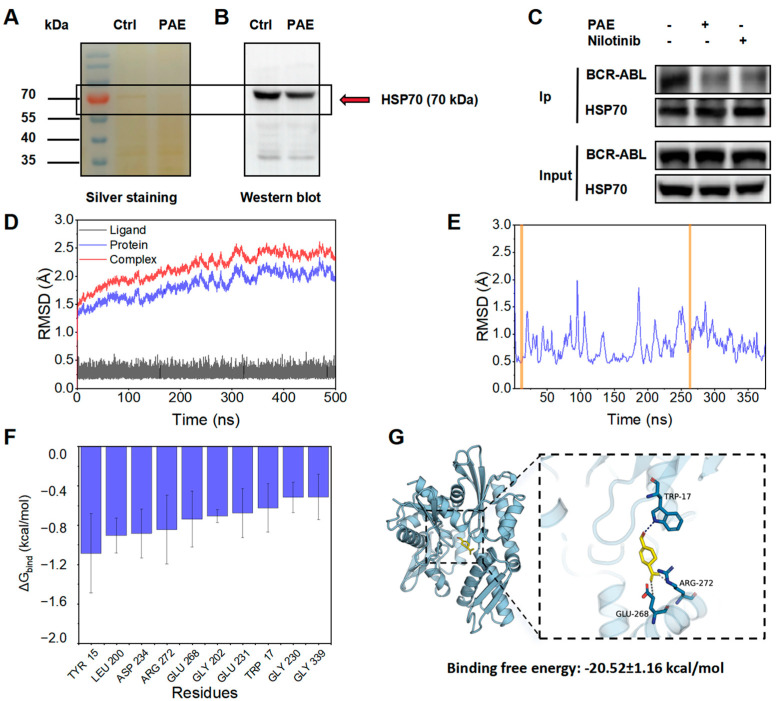
PAE targeted HSP70 and inhibited its stability. (**A**,**B**) HSP70 was validated as a target through silver staining and Western blotting. (**C**) The effect of PAE on the binding of HSP70 and BCR-ABL through immunoprecipitation. (**D**,**E**) RMSD analysis (Blue: the entire protein sequence; yellow: small molecule binding sites in (**E**)). (**F**) Top 10 amino acids contributing to PAE and HSP70 binding. (**G**) Molecular docking analysis of HSP70 and PAE.

Subsequently, we performed a DARTS assay to investigate the potential targets of PAE. Using silver staining, pronase-digested protein bands showed significant changes in enzymatic hydrolysis under PAE’s intervention (red arrow in Figure 6A, about 70 kDa). According to HPLC-MS/MS analysis, HSP70 was identified as the most likely candidate, and Western blotting further validated HSP70’s interaction with PAE (Figure 6B). Next, we further validated the binding of HSP70 and BCR-ABL proteins in K562 cells after PAE treatment using immunoprecipitation, with nilotinib as a control. The results showed that the interaction of the two proteins weakened after PAE was given (Figure 6C).

Next, we investigated the binding interaction between PAE and HSP70. The results showed that PAE exhibited minimal root mean square deviation (RMSD) fluctuations and remained stable in MD simulations, indicating its ability to maintain stable binding within the protein’s binding pocket (RMSD ≈ 0.3 Å, Figure 6D). Meanwhile, the RMSF values of most amino acids were less than 2 Å, indicating that HSP70 exhibits low flexibility, which provides a favorable foundation for its stable binding (Figure 6E). Figure 6F further revealed the top 10 amino acid residues contributing significantly to PAE–HSP70 binding: TYR 15, LEU 200, ASP 234, ARG 272, GLU 268, GLY 202, GLU 231, TRP 17, GLY 230, and GLY 339. Furthermore, Figure 6G demonstrated that PAE forms hydrogen bonding interactions with TRP 17 on HSP70 while engaging in hydrophobic interactions with ARG 272 and GLU 268. Furthermore, the calculated binding free energy of −20.52 ± 1.16 kcal/mol indicates a strong binding affinity between PAE and HSP70. All these results showed that HSP70 was the key target.

### 2.7. Effects of PAE on Toxicity in Zebrafish Embryos

**Figure 7:**
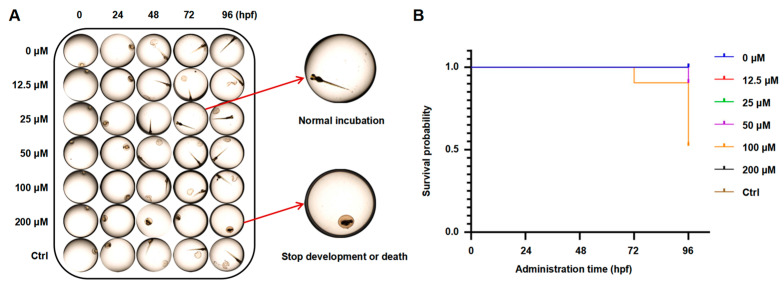
Developmental effects of PAE on zebrafish embryos. (**A**) Embryo morphology after PAE treatment for 0–96 hpf. (**B**) Survival probability statistics (n = 20).

Zebrafish are commonly used as aquatic models in toxicological research. We analyzed embryonic development at an early stage to assess PAE toxicity during embryonic development. As presented in Figure 7 and Figure S5 and Table 1, embryo malformation first appeared after exposure to 200 μM PAE for 72 hpf, with a 15% malformation rate; death and malformation began to occur simultaneously after PAE (200 μM) exposure for 96 hpf, with mortality and malformation rates of 10% and 35% (96 hpf) and 50% and 35% (120 hpf), respectively. In addition, embryo malformation began to occur after exposure to 100 μM PAE for 96 hpf, with a 10% malformation rate, and the malformed embryos died after 120 hpf. These results indicated that PAE might have toxic and teratogenic effects after long-term treatment at high concentrations.

## 3. Discussion

In malignant tumors, including CML, apoptosis and autophagy are involved in the abnormal activation and proliferation of cancer cells. Furthermore, the balance between these two factors can determine tumor progression or apoptosis. For example, peroxisome proliferator-activated receptor-γ inhibits tumor cell growth by regulating caspase 3 to induce apoptosis in CML cells [22]. Pyrimethamine attenuated the STAT5-Bcl-2 cascade by simultaneously targeting apoptotic and autophagic cell death mechanisms [23]. Moreover, CML cells can be affected by the crosstalk between apoptosis, autophagy, and necroptosis caused by tetrahydro benzimidazole TMQ0153 [24]. Proapoptotic and anti-apoptotic genes are known to regulate the mitochondrial membrane potential in most tumor cells. The apoptotic factor is further released, and caspase-related proteins are activated when the MMP changes [25]. Additionally, excessive ROS may increase mitochondrial membrane permeation and lead to MMP disappearance, an early event in the apoptotic process [26]. Our findings indicated that the MMP was reduced and ROS were increased by PAE treatment, indicating the significant inhibition of proliferation. Meanwhile, it induced caspase-dependent apoptosis. Additionally, autophagy may facilitate apoptosis and lead to autophagic cell death. Generally, apoptosis and autophagy work cooperatively to induce cell death.

In CML, chromosomes 9 and 22 translocate, generating a fusion oncogene with a constitutively active tyrosine kinase: the BCR-ABL oncogene [27]. JAK-STAT, PI3K/AKT, and MAPK/ERK pathways are mainly activated by this aberrant tyrosine kinase, resulting in malignant transformation. Meanwhile, chemotherapy insensitivity is further enhanced by these pathways [28]. In CML treatment, although imatinib was the first TKI, CML is becoming harder to treat due to the emergence of drug resistance [29]. Second-generation TKIs were then used to overcome imatinib resistance, including nilotinib, dasatinib, etc. Even though they show significant effects in imatinib-resistant patients, effects on patients with T315I mutations are limited [30]. Novel agents are therefore urgently needed to improve treatment outcomes for CML. Most anticancer agents affect their anticancer effect by blocking the AKT/mTOR/S6K1 signaling pathway, including in leukemic cells. Thus, inactivating key molecules could be effective in treating CML. Owing to their multi-target advantages, small-molecule compounds extracted from natural products are increasingly gaining attention in cancer therapy. For example, apigenin exerts significant inhibitory effects on K562 cells through multiple mechanisms, including cell apoptosis, ROS generation, and the BCR-ABL signaling pathway [31]. Caffeic acid enhances the anti-leukemic effects of imatinib in CML cells and induces apoptosis [32]. In our study, we found that PAE targeted HSP70, and the calculated binding free energy was −16.79 ± 2.01 kcal/mol, indicating a strong binding affinity between PAE and HSP70. Further results show that PAE suppressed the expression level of p-BCR-ABL and inhibited its downstream proteins, such as p-Akt, p-mTOR, and p-S6K1, in K562 cells. All these results show that HSP70 was the key target of PAE in CML treatment.

Zebrafish embryos have emerged as a preferred vertebrate model for pharmacological toxicity assessments due to their operational simplicity, optical transparency for in vivo observation, and heightened toxicological sensitivity. Our experimental data revealed that exposure to elevated PAE concentrations induced dose-dependent toxic effects, manifested through increased mortality rates and suppressed hatching success. These findings demonstrate that while PAE exhibits favorable safety profiles at therapeutic concentrations, its cytotoxic potential emerges at supraphysiological levels. These results indicate that PAE is safe at normal concentrations and can be used as a potential anticancer drug candidate.

## 4. Materials and Methods

### 4.1. Cells and Reagents

Human cervical cancer cells (Hela and CL-0101), human colon cancer cells (HCT-116 and CL-0096), human malignant melanoma cells (A375 and CL-0014), human pancreatic cancer cells (PANC-1 and CL-0184), human non-small-cell lung cancer cells (A549 and CL-0016), human embryonic kidney cells (293T and CL-0005), and human chronic myeloid leukemia cells (K562 and CL-0130) were purchased from Procell Life Science & Technology (Wuhan, China). Immortalized human cervical epithelium (H8, BFN607200572) and immortalized human keratinocytes (Hacat, BFN60803901) were purchased from ATCC Cell Bank (Shanghai, China).

Perillaldehyde (PAE) was purchased from Solarbio (SP8910, Beijing, China) and Z-Val-Ala-Asp(OMe)-fluoromethyl ketone (Z-VAD-fmk) was obtained from Shanghai Yuanye Bio-Technology (Shanghai, China). Antibodies against Bcl-2 (bs-0032R), survivin (bs-0615R) (bs-0050R), cleaved caspase-3 (bsm-61090R), caspase-3 (bs-0081R), cleaved caspase-9 (bs-3082R), caspase-9 (bs-0049R), LC3 (bs-8878R), p62 (bs-51287M), and Beclin-1 (bs-1353R) were acquired from Bioss (Beijing, China). AKT (# 9272S), P-AKT (# 86758S), BCR-ABL (# 3908), p-BCR-ABL (# 2861), RPS6-Kb1 (# 34475), p-RPS6-Kb1 (# 9234), HSP70 (# 4872), mTOR (#2983), and P-mTOR (#2974) were purchased from CST (Beverly, MA, USA).

### 4.2. Cell Viability Assessment

Cells (5 × 10^3^ cells/well in a 96-well plate) were incubated with PAE at concentrations ranging from 0 to 200 μM for different durations (24, 48, and 72 h). After incubation with the CCK-8 solution for an additional 2 h, the absorbance was measured at 450 nm.

### 4.3. Cell Cycle Analysis

Cells (4 × 10^5^ cells/well) were cultured with PAE (0–200 μM) for 24 h, then were collected, washed, and incubated overnight in 75% ethanol at –20 °C. In the cell cycle assay kit (CA1510, Solarbio, Beijing, China), DNA content was measured from resuspended cells in PBS.

### 4.4. Monodansylcadaverine (MDC) Staining

Cells (2 × 10^5^ cells/well) were cultured in 6-well plates and treated with PAE for 24 h. Positive Ctrl cells were pretreated with Earle’s Balanced Salt Solution (C0213-500, Beyotime, Beijing, China) for 30 min. Finally, cells were stained with MDC (R22204, Yuanye, Shanghai, China) for 30 min at 37 °C and photographed.

### 4.5. Measurement of the Mitochondrial Membrane Potential (MMP)

Cells (2 × 10^5^ cells/well) were incubated with PAE (0–200 μM) for 24 h. Carbonyl cyanide m-chlorophenyl hydrazine (CCCP) was used as a positive control, and then the cells were stained with JC–1 (M8650, Solarbio, Beijing, China) for 20 min at 37 °C. The MMP was analyzed using laser scanning confocal microscopy (Nikon C2, Nikon Corporation, Tokyo, Japan).

### 4.6. Measurement of Reactive Oxygen Species (ROS) Generation

Cells (2 × 10^5^ cells/well) were treated with PAE (0–200 μM) for 24 h. Then, cells were incubated with 2′,7′-dichlorodihydrofluorescein diacetate (DCFH–DA) (S0033S, Beyotime, China) and Hoechst 33342 (C0030, Solarbio, Beijing, China) for 30 min at 37 °C. Finally, laser scanning confocal microscopy (Nikon C2, Nikon Corporation, Japan) was performed.

### 4.7. Drug Affinity Responsive Target Stability (DARTS)

Cells were collected and lysed with M-PER lysis buffer (containing 1% protease inhibitors) on ice for 20 min. The lysate was diluted to a concentration of 5 μg/μL using 1× PBS solution. Then, the treatment group was supplemented with 100 μM PAE for 1 h, and the control group was treated with an equal volume of 1× TNC buffer. The samples were incubated at 37 °C for 1 h. Then, pronase E was added and incubated for 10 min. Finally, 5× loading buffer was added and boiled for another 10 min. All samples were eluted with 1× SDS sample buffer for further analysis.

### 4.8. Coimmunoprecipitation (Co-IP)

The HSP70 antibody was added to the cell lysate and incubated overnight at 4 °C. Subsequently, resuspended protein A/G beads (B23201, bimake, Beijing, China) were directly added (incubated for 6 h at 4 °C). To collect pellets, the solution was centrifuged (4 °C, 2500 rpm, 5 min). After treatment with PAE and nilotinib, proteins were extracted from K562 cells and incubated with beads (4 °C overnight). Beads were collected and washed with 1× PBST buffer.

### 4.9. Molecular Dynamics (MD) Assay and Molecular Docking

The small molecule–protein complex obtained through docking was used as the initial structure for the all-atom molecular dynamics simulation, which was carried out using AMBER 22 software. Before the simulation, the system was energy-optimized, including 2500 steps of steepest descent and 2500 steps of conjugate gradient. Then, the system was heated at a constant volume and a constant heating rate for 200 ps, gradually raising the system temperature from 0 K to 298.15 K. Under the condition of maintaining the system temperature at 298.15 K, a 500 ps NVT (isothermal-isochoric) ensemble simulation was conducted to evenly distribute the solvent molecules in the solvent box. During the simulation, the non-bonded cutoff distance was set to 10 Å, the particle mesh Ewald (PME) method was used to calculate the long-range electrostatic interactions, the SHAKE method was used to restrict the bond lengths of hydrogen atoms, and the Langevin algorithm was used for temperature control, with the collision frequency γ set to 2 ps^−1^. The system pressure was 1 atm, the integration step was 2 fs, and the trajectory was saved every 10 ps for subsequent analysis.

The target compounds were downloaded from the PubChem database, and the crystal structure of the target protein was obtained from the PDB database (PDB ID: 6B1N). Before docking, the charge state of the compounds at pH 7.4 was calculated using the LigPrep module, and energy minimization was performed in the OPLS4 force field. Additionally, the Protein Preparation Wizard in Maestro was used to optimize the downloaded protein, including removing ligand molecules and water molecules, adding hydrogen atoms, and optimizing the structure using the OPLS4 force field to eliminate intermolecular clashes. The Induce Fit Docking module in Maestro 13.0 was used for molecular docking. The centroid of the co-crystallized ligand in the crystal structure was selected as the center of the active pocket, and a box file with a volume of 20 × 20 × 20 Å^3^ was generated. Molecular docking was completed using SP (standard) precision.

### 4.10. Acute Toxicity Test of Zebrafish Embryos

The Chinese Academy of Sciences (Wuhan, China) provided AB wild-type adult zebrafish. Males and females were placed equally in separate spawning boxes during breeding. The eggs were mixed and laid early in the morning. The embryos were divided into 7 groups and placed in 96-well plates (n = 20). PAE was diluted with a Holt buffer culture solution to a final concentration of 0–160 μM (1% DMSO as the Ctrl). Finally, the mortality and malformation rates at 24, 48, 72, 96, and 120 hpf were photographed and recorded, respectively. The zebrafish were euthanized by overexposure to tricaine mesylate.

### 4.11. Statistical Analysis

Data were expressed as mean values ± SD. Significant differences between the two groups were determined using *t*-tests or analysis of variance (ANOVA) for multiple groups. Differences were considered statistically significant at *p* < 0.05.

## 5. Conclusions

This study demonstrates that PAE exerts significant anti-CML effects in K562 cells by modulating multiple cellular pathways. Specifically, PAE effectively arrests the cell cycle, induces apoptosis, and triggers autophagy by targeting HSP70. Meanwhile, the inactivation of the BCR-ABL signaling pathway was partially responsible for the PAE-induced inhibition of cell proliferation. Collectively, these findings provide a new understanding of PAE’s anticancer mechanisms and highlight its potential as a novel therapeutic agent for the treatment of CML.

## Figures and Tables

**Table 1:** The mortality and malformation rates after treatment with PAE for 24–120 hpf.

	PAE (μM)	Survival Number	Number of Deaths	Number of Deformities	Mortalities	Malformation Rates
24 hpf	0	20	0	0	0.00%	0.00%
	12.5	20	0	0	0.00%	0.00%
	25	20	0	0	0.00%	0.00%
	50	20	0	0	0.00%	0.00%
	100	20	0	0	0.00%	0.00%
	200	20	0	0	0.00%	0.00%
	Ctrl	20	0	0	0.00%	0.00%
48 hpf	0	20	0	0	0.00%	0.00%
	12.5	20	0	0	0.00%	0.00%
	25	20	0	0	0.00%	0.00%
	50	20	0	0	0.00%	0.00%
	100	20	0	0	0.00%	0.00%
	200	20	0	0	0.00%	0.00%
	Ctrl	20	0	0	0.00%	0.00%
72 hpf	0	20	0	0	0.00%	0.00%
	12.5	20	0	0	0.00%	0.00%
	25	20	0	0	0.00%	0.00%
	50	20	0	0	0.00%	0.00%
	100	20	0	0	0.00%	0.00%
	200	20	0	3	0.00%	15.00%
	Ctrl	20	0	0	0.00%	0.00%
96 hpf	0	20	0	0	0.00%	0.00%
	12.5	20	0	0	0.00%	0.00%
	25	20	0	0	0.00%	0.00%
	50	20	0	0	0.00%	0.00%
	100	20	0	2	0.00%	10.00%
	200	18	2	7	10.00%	35.00%
	Ctrl	20	0	0	0.00%	0.00%
120 hpf	0	20	0	0	0.00%	0.00%
	12.5	20	0	0	0.00%	0.00%
	25	20	0	0	0.00%	0.00%
	50	20	0	0	0.00%	0.00%
	100	18	2	2	10.00%	10.00%
	200	10	10	7	50.00%	35.00%
	Ctrl	20	0	0	0.00%	0.00%

## Data Availability

Data are contained within the article.

## Supplementary Materials

The following supporting information can be downloaded at: https://www.mdpi.com/article/10.3390/molecules30112294/s1.
