# Supplementary material for: Unlocking the Potential of Perillaldehyde: A Novel Mechanism for Chronic Myeloid Leukemia by Targeting HSP70
Source: Molecules. 2025 May 23;30(11):2294. doi: 10.3390/molecules30112294 (PMC12155963; doi:10.3390/molecules30112294)
Supplement: Supplementary file 1 [file molecules-30-02294-s001.zip › molecules-3628327-supplementary.pdf]

Unlocking the potential of perillaldehyde: a novel mechanism for Chronic Myeloid Leukemia by targeting HSP70

Miaomiao Zhang [1,†], Jinfeng Wang [1], Rongsong Jiang [1], Ming Liu [1,2] and Weiyi Zhang [1,3,4,5,*,†]

1 School of Pharmacy, Xinjiang Medical University, Urumchi 830017, China; zmm@xjmu.edu.cn (M.Z.); wjf@xjmu.edu.cn (J.W.); jrs@stu.xjmu.edu.cn (R.J.); lmouc@ouc.edu.cn (M.L.)
2 Key Laboratory of Marine Drugs, Chinese Ministry of Education, School of Medicine and Pharmacy, Ocean University of China, Qingdao 266003, China
3 Xinjiang Key Laboratory of Natural Medicines Active Components and Drug Release Technology, Urumchi 830017, China
4 Xinjiang Key Laboratory of Biopharmaceuticals and Medical Devices, Urumchi 830017, China
5 Engineering Research Center of Xinjiang and Central Asian Medicine Resources, Ministry of Education, Urumchi 830017, China
* Correspondence: zwy@xjmu.edu.cn; Tel.: +86-0991-4362505
† These authors contributed equally to this work.

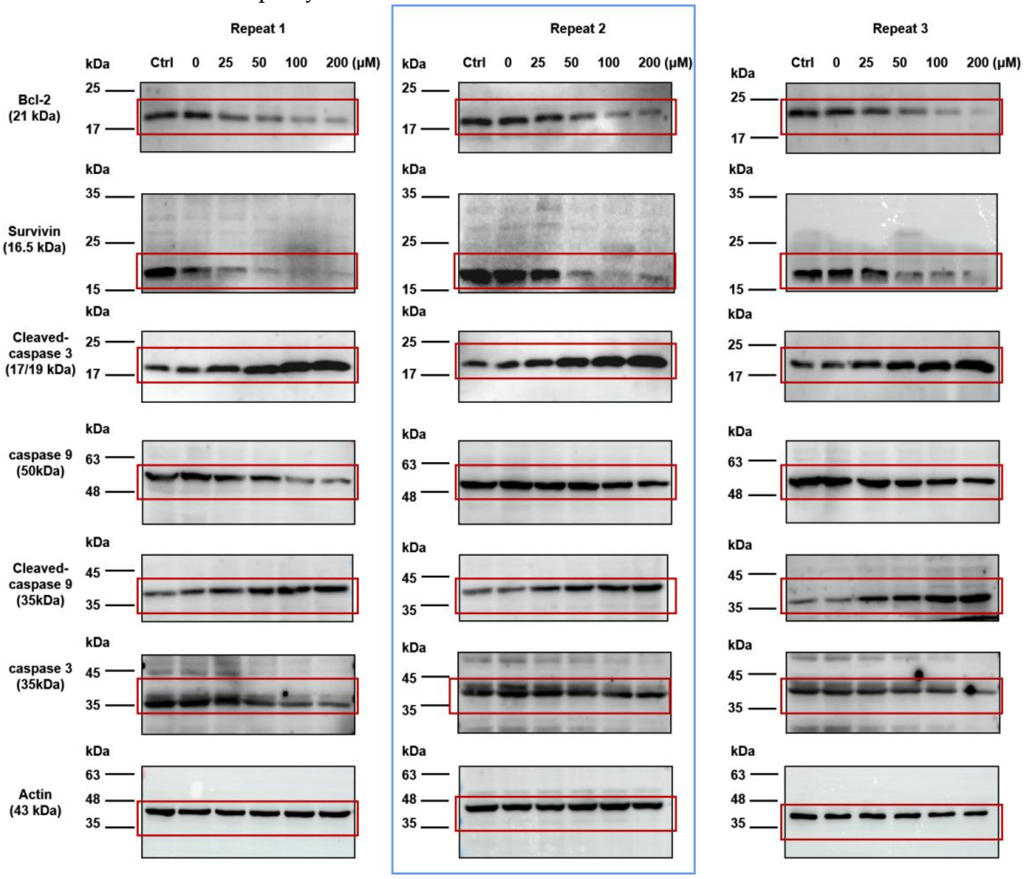

		Repeat 1			Repeat 2			Repeat 3				
Survivin	PAE (μM)	intden	Normalization to Actin (with same group)	Quadratic normalization to 0 (in each group)	intden	Normalization to Actin (with same group)	Quadratic normalization to 0 (in each group)	intden	Normalization to Actin (with same group)	Quadratic normalization to 0 (in each group)	The mean value of the three normalized groups	The standard deviation of the three normalized groups
	Ctrl	5982732	0.082358082	1	3118966	0.097954417	1	67830661	1.446354544	1	1	0
	0	3505212	0.049945962	0.60644882	3235934	0.107167492	1.09405472	65154793	1.571823895	1.086748683	0.929084074	0.279434205
	25	2157382	0.033453246	0.406192628	2174814	0.073460685	0.749947649	63839179	1.445886285	0.999676249	0.718605509	0.297980619
	50	2063078	0.030566953	0.371146977	1844568	0.059289326	0.605274654	37909006	0.905108587	0.625786112	0.534069248	0.141467062
	100	1907787	0.026239068	0.318597363	1612016	0.047246198	0.482328411	33150709	0.876459038	0.605978003	0.468967926	0.144155419
	200	1386344	0.019558103	0.237476423	1320495	0.043073333	0.439728341	23959799	0.722783504	0.499727751	0.392310838	0.137405439
Bcl-2												
	Ctrl	58157614	0.800595708	1	34924266	1.096833409	1	61731374	1.316299325	1	1	0
	0	44375548	0.632309666	0.78979897	31868707	1.055426164	0.962248373	50458631	1.217286991	0.924779773	0.892275706	0.09070328
	25	36490772	0.565840806	0.706774718	29914556	1.01045136	0.921244149	44678281	1.011913291	0.768756218	0.798925028	0.110371654
	50	31253482	0.463057492	0.578391175	22812973	0.73326968	0.66853332	38965892	0.930342607	0.706786511	0.651237002	0.065922018
	100	25383002	0.349109368	0.436062002	23662097	0.693506842	0.632280925	28433839	0.751751499	0.571109842	0.546484256	0.100400603
	200	22317992	0.314855174	0.39327612	24996399	0.815359562	0.743375936	26592370	0.802198982	0.609435078	0.582029045	0.176651601
caspase 3												
	Ctrl	71832606	0.988845177	1	62517784	1.963436945	1	61908571	1.3200777	1	1	0
	0	75556779	1.076612771	1.08875767	63165147	2.091900019	1.065427654	71937830	1.735460969	1.314665772	1.156283699	0.13765803
	25	62942449	0.976011307	0.987021356	57047855	1.926957655	0.981420697	63837384	1.44584563	1.095273127	1.021238393	0.064177085
	50	59391552	0.879956452	0.889882939	56520859	1.816730866	0.925280983	60081140	1.434486458	1.0866682	0.967277374	0.104899392
	100	52907617	0.727673768	0.735882406	47570310	1.394227041	0.710095144	53761075	1.421368698	1.076731088	0.840902879	0.204639814
	200	45515801	0.642122528	0.649366092	44945378	1.466076922	0.746689078	52990660	1.598543247	1.210946331	0.8690005	0.300105239
C-caspase 3												
	Ctrl	45368297	0.624538412	1	51789075	1.626490524	1	40046073	0.853903217	1	1	0
	0	41965759	0.597972448	0.957463042	47555428	1.574938165	0.968304544	33860322	0.816861827	0.95662109	0.960796226	0.006516008
	25	66470860	1.030724289	1.650377734	57020084	1.926019608	1.184156674	43668678	0.989046908	1.158265818	1.330933409	0.27694962
	50	70966346	1.051450786	1.68356464	69740695	2.241651586	1.378213739	50238097	1.19947574	1.404697529	1.488825303	0.169168274
	100	75296880	1.035608245	1.658197839	77596366	2.274253663	1.39825817	58078384	1.535512396	1.798227674	1.618227894	0.202958366
	200	86916545	1.226191132	1.963355831	99274475	3.238242134	1.990938212	79201941	2.389246104	2.798029163	2.250774402	0.474137139
caspase 9												
	Ctrl	56998243	0.784635847	1	62327559	1.957462729	1	61429692	1.309866554	1	1	0
	0	58897146	0.839228728	1.069577348	61438666	2.034722512	1.039469351	61573660	1.485431012	1.134032324	1.081026341	0.048309922
	25	47120928	0.730676344	0.931229878	57665942	1.947835346	0.995081703	54056411	1.224317488	0.934688717	0.953666766	0.035908058
	50	43559335	0.645383335	0.822525936	45239772	1.4541267	0.742863033	53372476	1.274311607	0.972856054	0.846081675	0.116791918
	100	28114553	0.386678211	0.492812319	44266579	1.297398765	0.662796152	45272947	1.196954297	0.913798657	0.689802376	0.211788518
	200	26792937	0.377986283	0.48173466	42068841	1.372246929	0.70103349	36444497	1.099403264	0.839324633	0.674030928	0.180317778
C-caspase 9												
	Ctrl	10571140	0.145521949	1	9662533	0.30346204	1	36389238	0.775928451	1	1	0
	0	10817267	0.154135843	1.059193091	9926299	0.328738649	1.083294136	35754780	0.862564594	1.111654809	1.084714012	0.026259665
	25	15708393	0.243580754	1.673842031	13382725	0.452040561	1.489611553	46786959	1.059672499	1.365683263	1.509712282	0.155059621
	50	15923928	0.235931925	1.621280688	16207030	0.520937087	1.716646625	49809085	1.189232726	1.532657714	1.623528342	0.092015047
	100	19981505	0.274818974	1.88850532	17534054	0.51390147	1.693462119	59704387	1.578501673	2.034339211	1.872102217	0.171029513
	200	19432017	0.274140751	1.883844696	18436330	0.60137614	1.981717845	65365331	1.971843877	2.541270235	2.135610925	0.354703249
Actin												
	Ctrl	72642925	1		31840994	1		46897672	1			
	0	70180088	1		30195108	1		41451713	1			
	25	64489467	1		29605142	1		44152282	1			
	50	67493740	1		31111300	1		41883379	1			
	100	72707880	1		34119486	1		37823455	1			
	200	70883358	1		30656903	1		33149344	1			

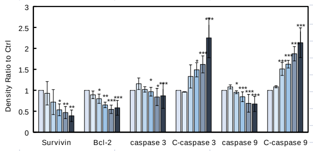

Supplement Figure S1 Original unedited bands for evaluating PAE treatment (0-200 μM) affected apoptotic proteins measured by western blotting in Fig. 2C/D. Image software was used for sensitivity analysis, and the relative quantitative statistics and normalized the intensity value against "Ctrl" group were adopted. Western blotting was performed 3 times. The "Repeat 2" was used in the manuscript.

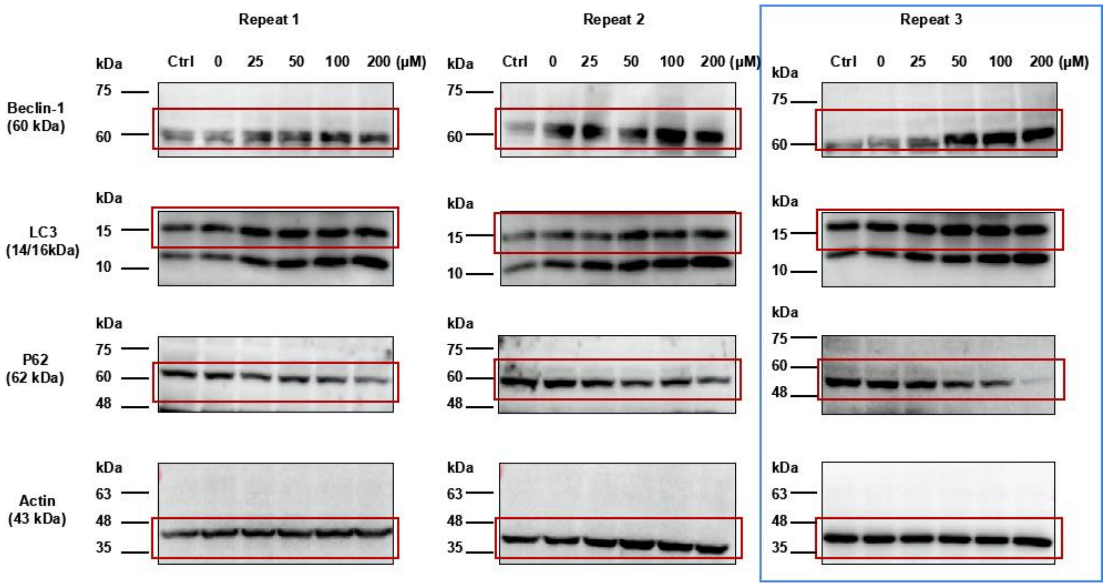

		Repeat 1			Repeat 2			Repeat 3				
Beclin-1	PAE (μM)	intden	Normalization to Actin (with same group)	Quadratic normalization to 0 (in each group)	intden	Normalization to Actin (with same group)	Quadratic normalization to 0 (in each group)	intden	Normalization to Actin (with same group)	Quadratic normalization to 0 (in each group)	The mean value of the three normalized groups	The standard deviation of the three normalized groups
	Ctrl	9830358	0.491267059	1	14644978	1.294457592	1	9269102	6.004984555	1	1	0
	0	7762887	0.334017384	0.679909996	22171206	1.603177285	1.238493478	11075077	6.92597482	1.153370963	1.023924812	0.300950338
	25	19430700	0.932866024	1.898897976	36431473	3.007624759	2.32346334	10441662	6.648690467	1.107195265	1.77651886	0.617300165
	50	23040983	0.970056795	1.97460175	30551261	2.347661572	1.813625712	12012217	7.29377751	1.214620528	1.667615997	0.400477206
	100	24376846	1.079162709	2.196692592	29512550	2.520279408	1.946977193	22923761	13.63801705	2.271116092	2.138261959	0.169785484
	200	19459296	0.827442313	1.684302455	25682428	2.247148828	1.73597717	21779683	13.36209662	2.225167524	1.881815716	0.298471803
LC3												
	Ctrl	33078443	1.653078088	1	42260296	3.735352896	1	38410027	24.88392283	1	1	0
	0	42485075	1.828025271	1.105831167	58662738	4.241842734	1.135593571	43324088	27.09340464	1.088791539	1.110072092	0.023687478
	25	49921583	2.396730362	1.449859132	55738954	4.601566841	1.231896147	51620293	32.86903464	1.320894413	1.334216564	0.10959049
	50	51459662	2.166521922	1.310598657	64939504	4.990169736	1.335929931	54064197	32.82759745	1.319229193	1.32191926	0.01287811
	100	57077971	2.52684116	1.52856733	65599710	5.602009935	1.499727091	63706770	37.90102399	1.523112905	1.517135776	0.015321044
	200	65439387	2.78259387	1.683280354	72773641	6.367513307	1.704661777	66694854	40.91809247	1.644358598	1.677433577	0.030573795
P62												
	Ctrl	54998	1.625428538	1	47175	1.989651711	1	48764	0.031591741	1	1	0
	0	42366	1.090473862	0.670883915	29251	1.768420733	0.888809194	36921	0.023089132	0.730859764	0.763517624	0.112573348
	25	17771	0.456368772	0.280768279	33155	0.745537315	0.374707448	33506	0.021334824	0.675329175	0.443601634	0.206105269
	50	13788	0.406989787	0.250389222	23901	0.868775734	0.436647142	12725	0.007726577	0.244575853	0.310537406	0.109252909
	100	21705	0.685760324	0.421895093	10092	0.679585914	0.341560239	10983	0.006534108	0.206829644	0.323428325	0.108673187
	200	12121	0.597028978	0.367305584	9300	0.99135122	0.498253646	8450	0.005184176	0.164099103	0.343219444	0.168374349
Actin												
	Ctrl	20010212	1		11313602	1		1543568	1			
	0	23240967	1		13829541	1		1599064	1			
	25	20829036	1		12113038	1		1570484	1			
	50	23752200	1		13013486	1		1646913	1			
	100	22588666	1		11710031	1		1680872	1			
	200	23517405	1		11428895	1		1629960	1			

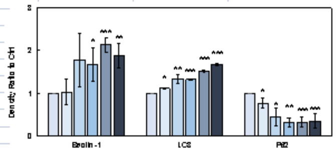

Supplement Figure S2 Original unedited bands for evaluating PAE treatment (0-200 μM) affected autophagy-related proteins measured by western blotting in Fig. 3C/D. Image software was used for sensitivity analysis, and the relative quantitative statistics and normalized the intensity value against "Ctrl" group were adopted. Western blotting was performed 3 times. The "Repeat 3" was used in the manuscript.

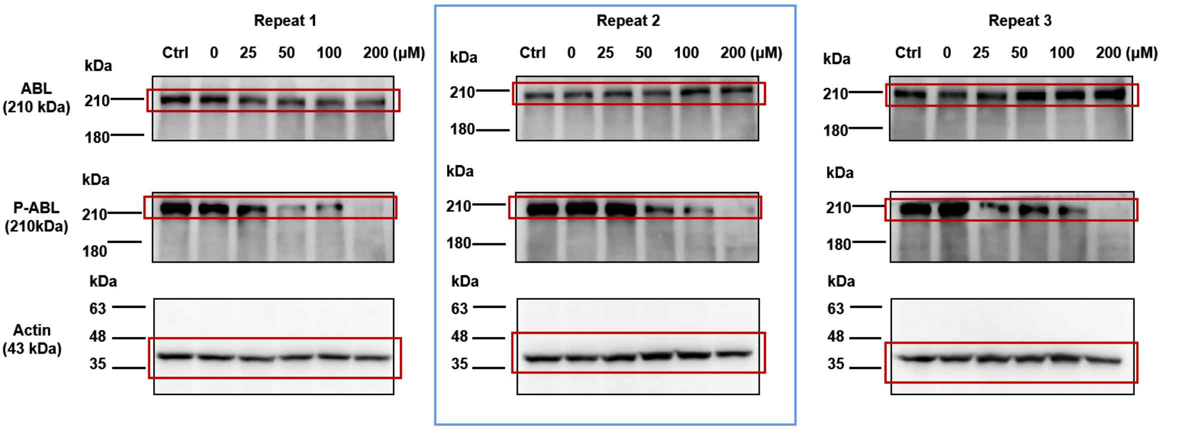

		Repeat 1			Repeat 2			Repeat 3				
ABL	PAE (μM)	intden	Normalization to Actin (with same group)	Quadratic normalization to 0 (in each group)	intden	Normalization to Actin (with same group)	Quadratic normalization to 0 (in each group)	intden	Normalization to Actin (with same group)	Quadratic normalization to 0 (in each group)	The mean value of the three normalized groups	The standard deviation of the three normalized groups
	Ctrl	44761150	1.768574185	1	59124602	2.284328021	1	36343488	1.424928995	1	1	0
	0	43521640	1.675205969	0.947207069	50502004	2.090268881	0.915047603	38537740	1.708496246	1.199004479	1.020419717	0.15549259
	25	36926330	1.593068682	0.90076441	50887002	1.874209454	0.82046424	41167334	1.56111888	1.095576612	0.938935087	0.141472452
	50	34641962	1.577474593	0.891947087	59379431	2.25982295	0.989272525	41141426	1.50418655	1.055622109	0.97894724	0.082324582
	100	35658935	1.624419638	0.918491094	57865972	2.30782406	1.010285755	51696993	1.989077253	1.395913242	1.108230031	0.253333353
	200	34032405	1.669495484	0.943978205	67904100	2.890992615	1.26557683	54147705	2.483625495	1.742981934	1.317512323	0.402025759
P-ABL												
	Ctrl	71752156	1.603000727	1	74807594	1.265253236	1	64178258	1.765880534	1	1	0
	0	62762563	1.442100137	0.899625379	76065969	1.506197041	1.19043129	71980246	1.867785864	1.057707941	1.04925487	0.145587122
	25	56993664	1.543442416	0.962845737	78545023	1.543518382	1.219928421	47466819	1.153021447	0.652944197	0.945239451	0.283901856
	50	32620579	0.941649292	0.587429111	51398264	0.865590376	0.684124214	61263611	1.489097899	0.843260838	0.704938054	0.129179643
	100	37071630	1.039616859	0.648544222	39723019	0.686465942	0.542552212	48053908	0.929530041	0.52638331	0.572493248	0.066356396
	200	25407457	0.746566603	0.46573067	27855886	0.410223919	0.324222778	26213074	0.48410314	0.274142633	0.354698694	0.099363374
Actin												
	Ctrl	25309173	1		25882711	1		25505473	1			
	0	25979874	1		24160530	1		22556526	1			
	25	23179371	1		27151182	1		26370403	1			
	50	21960393	1		26276143	1		27351279	1			
	100	21951800	1		25073823	1		25990440	1			
	200	20384844	1		23488161	1		21801880	1			

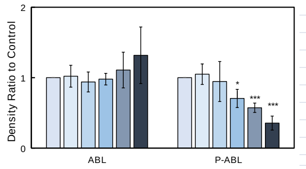

Supplement Figure S3 Original unedited bands for evaluating PAE treatment (0-200 μM) affected ABL and P-ABL proteins measured by western blotting in Fig. 5A/B. Image software was used for sensitivity analysis, and the relative quantitative statistics and normalized the intensity value against "Ctrl" group were adopted. Western blotting was performed 3 times. The "Repeat 2" was used in the manuscript.

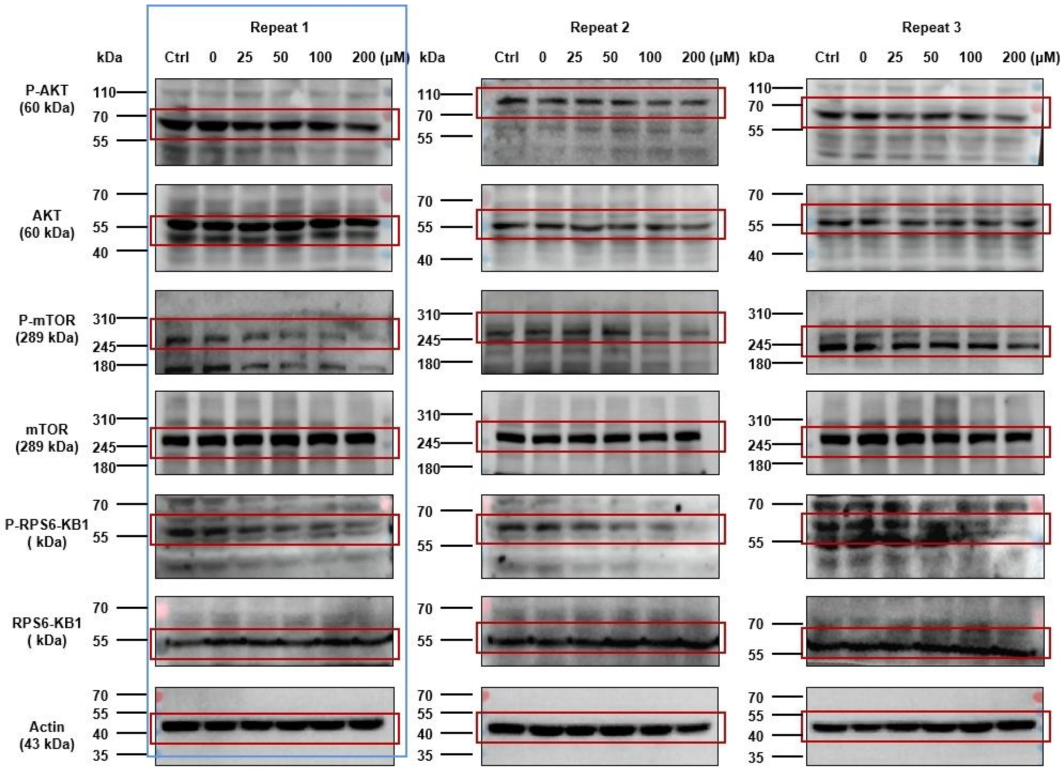

		Repeat 1				Repeat 2				Repeat 3					
	PAE (μM)	intden	Normalization to Actin (with same group)	Normalization to AKT (with same group)	Quadratic normalization to Ctrl (in each group)	intden	Normalization to Actin (with same group)	Normalization to AKT (with same group)	Quadratic normalization to Ctrl (in each group)	intden	Normalization to Actin (with same group)	Normalization to AKT (with same group)	Quadratic normalization to Ctrl (in each group)	The mean value of the three normalized groups	The standard deviation of the three normalized
P-AKT	Ctrl	111672	4.703634037	6.550854984	1	97039	3.790394343	4.494571872	1	21890	0.743316345	0.818883572	1	1	0
	0	109941	5.297518889	4.625855004	0.706145231	91735	3.722181462	3.9507055	0.878994844	19362	0.626272172	0.771757428	0.94245074	0.842530271	0.122300132
	25	102689	4.250021076	4.229267779	0.645605465	92500	3.199645813	4.238816069	0.943096737	18569	0.673293661	0.728927844	0.890148331	0.826283511	0.158695623
	50	99889	3.979015636	4.874424238	0.744089779	91327	3.278845935	3.898750459	0.867435335	16864	0.524369041	0.66162287	0.807957191	0.806494102	0.061685793
	100	87388	3.259292768	4.037720577	0.616365434	77045	2.60542449	3.491238769	0.776767814	12699	0.449944762	0.466668161	0.569883409	0.654338886	0.108543892
	200	68605	2.567067507	4.052875022	0.618678788	59563	2.170476554	2.88592623	0.642091463	11080	0.425136094	0.43519134	0.531444708	0.597404986	0.058310437
AKT	Ctrl	17047	0.718018342	1		21590	0.843327118	1		26731	0.907719206	1			
	0	23767	1.145197782	1		23220	0.942156144	1		25088	0.811488364	1			
	25	24281	1.004907066	1		21822	0.754844221	1		25475	0.923676693	1			
	50	20492	0.816304745	1		23425	0.840999179	1		25489	0.79254975	1			
	100	21643	0.807211075	1		22068	0.746275079	1		27211	0.96416426	1			
	200	16928	0.633394194	1		20639	0.752090102	1		25459	0.976894654	1			
Actin															
	Ctrl	23742	1			25601	1			29449	1				
	0	20753	1			24645	1			30916	1				
	25	24162	1			28910	1			27580	1				
	50	25104	1			27853	1			32161	1				
	100	26812	1			29571	1			28223	1				
	200	26725	1			27442	1			26062	1				

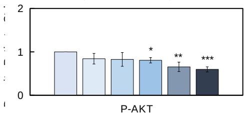

	PAE (μM)	intden	Normalization to Actin (with same group)	Normalization to mTOR (with same group)	Quadratic normalization to Ctrl (in each group)	intden	Normalization to Actin (with same group)	Normalization to mTOR (with same group)	Quadratic normalization to Ctrl (in each group)	intden	Normalization to Actin (with same group)	Normalization to mTOR (with same group)	Quadratic normalization to Ctrl (in each group)	The mean value of the three normalized groups	The standard deviation of the three normalized groups
P-mTOR	Ctrl	31456	1.324929533	0.32875567	1	45693	1.784795943	0.603670137	1	55126	1.871928014	0.670991772	1	1	0
	0	30159	1.453209908	0.293792741	0.893650719	46218	1.875318163	0.606074117	1.003982274	53258	1.722650657	0.556853232	0.829895769	0.909176254	0.08807559
	25	17851	0.73880183	0.17035185	0.518171595	39692	1.372969438	0.53264983	0.882352459	48654	1.764109285	0.510385196	0.760643003	0.720389019	0.185397445
	50	8645	0.34436905	0.081109735	0.246717372	38825	1.393909786	0.526862168	0.872765001	36375	1.131019191	0.429822281	0.640577574	0.586686649	0.316483937
	100	8568	0.319559312	0.07863797	0.239198825	11236	0.379967296	0.148583066	0.246132874	33259	1.178454775	0.429392171	0.639936567	0.375089422	0.229390558
	200	2661	0.099569226	0.027530054	0.083740166	10524	0.383497006	0.134648601	0.223049962	19684	0.755286574	0.247208791	0.368422985	0.225071038	0.14235217
mTOR	Ctrl	95682	4.030134397	1		75692	2.956574848	1		82156	2.7897928	1			
	0	102654	4.94637786	1		76258	3.09420599	1		95641	3.093545222	1			
	25	104789	4.336916977	1		74518	2.577621096	1		95328	3.456427219	1			
	50	106584	4.245717854	1		73691	2.645682062	1		84628	2.631364731	1			
	100	108955	4.063677042	1		75621	2.557271886	1		77456	2.744471965	1			
	200	96658	3.616746414	1		78159	2.848132125	1		79625	3.055257747	1			

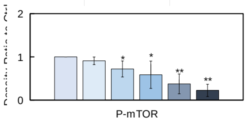

	PAE (μM)	intden	Normalization to Actin (with same group)	Normalization to RPS6-KB1 (with same group)	Quadratic normalization to Ctrl (in each group)	intden	Normalization to Actin (with same group)	Normalization to RPS6-KB1 (with same group)	Quadratic normalization to Ctrl (in each group)	intden	Normalization to Actin (with same group)	Normalization to RPS6-KB1 (with same group)	Quadratic normalization to Ctrl (in each group)	The mean value of the three normalized groups	The standard deviation of the three normalized groups
P-RPS6-KB1	Ctrl	25078	1.056289877	1.894648321	1	26273.59	1.02626212	3.132775528	1	26883.6	0.912893783	3.915424497	1	1	0
	0	22681	1.092900584	1.704932731	0.899867649	24337	0.98748595	2.838457648	0.906052037	25854.5	0.836275087	3.635811476	0.928586793	0.91150216	0.015115395
	25	23684	0.980193936	1.810862912	0.955777857	20049.25	0.693515117	2.372960021	0.757462512	21734.5	0.78805471	2.990899388	0.763876149	0.825705506	0.112691597
	50	19623	0.781664884	1.445441122	0.762907346	17902.37	0.642737565	2.111044159	0.67385746	19419.2	0.603807102	2.726843757	0.696436302	0.711067036	0.046292703
	100	18830	0.702298201	1.456999856	0.769008074	13904.71	0.470214974	1.574355152	0.502543236	16038.2	0.568276659	2.369027898	0.605050078	0.625533796	0.134408201
	200	8034	0.300619548	0.612438452	0.323246507	8322.196	0.303262756	0.981761166	0.313383821	14035.6	0.538555703	1.96677555	0.502314769	0.379648366	0.106346617
RPS6-KB1	Ctrl	13236	0.557512371	1		8386.681	0.327588782	1		6866.08	0.233153208	1			
	0	13303	0.641022701	1		8574.024	0.347895256	1		7111.08	0.230010575	1			
	25	13079	0.541285555	1		8449.045	0.292257396	1		7266.87	0.263484192	1			
	50	13576	0.54077947	1		8480.338	0.304464292	1		7121.49	0.221430766	1			
	100	12924	0.482016658	1		8832.004	0.298671474	1		6769.96	0.239877572	1			
	200	13118	0.49085675	1		8476.803	0.308896671	1		7136.37	0.273826723	1			

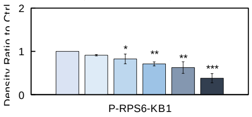

Supplement Figure S4 Original unedited bands for evaluating PAE treatment (0-200 μM) affected related signaling pathways proteins measured by western blotting in Fig. 5C/D. Image software was used for sensitivity analysis, and the relative quantitative statistics and normalized the intensity value against “Ctrl” group were adopted. Western blotting was performed 3 times. The “Repeat 1” was used in the manuscript.

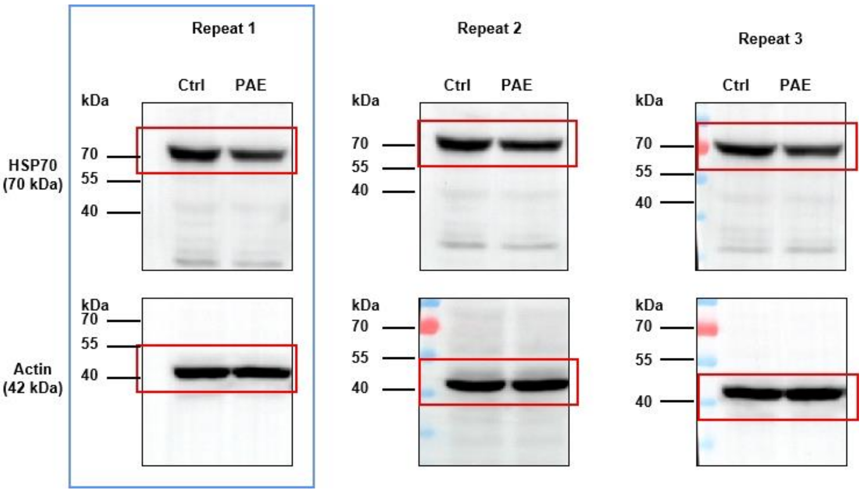

Supplement Figure S5 Original unedited bands for evaluating PAE treatment affected related signaling pathways proteins measured by western blotting in Fig. 6B. Image software was used for sensitivity analysis, and the relative quantitative statistics and normalized the intensity value against “Ctrl” group were adopted. Western blotting was performed 3 times. The “Repeat 1” was used in the manuscript.

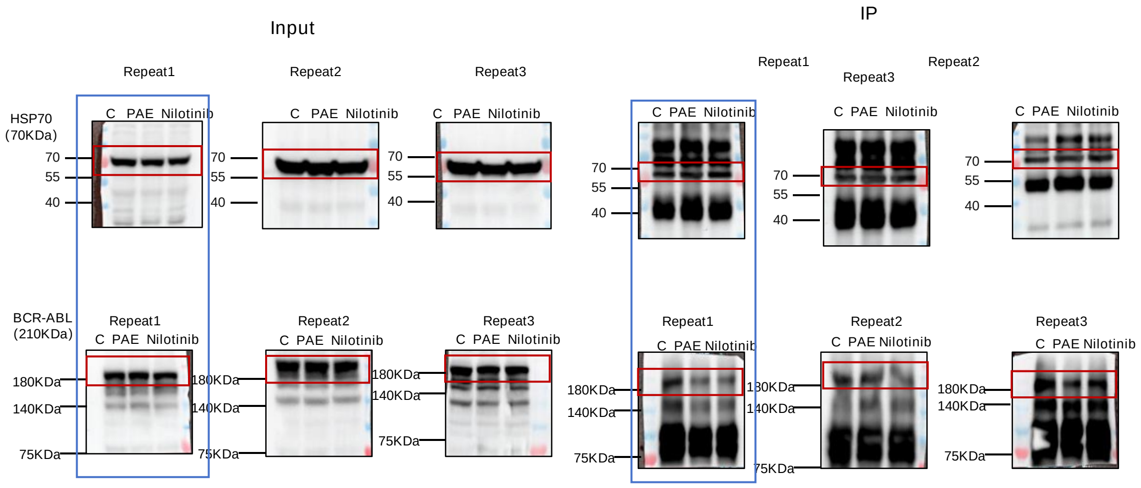

		Repeat 1			Repeat 2			Repeat 3				
BCR-ABL(IP)		intden	Normalization to Actin (with same group)	Quadratic normalization to Ctrl (in each group)	intden	Normalizatio n to Actin (with same group)	Quadratic normalization to Ctrl (in each group)	intden	Normalizatio n to Actin (with same group)	Quadratic normalizatio n to Ctrl (in each group)	The mean value of the three normalized groups	The standard deviation of the three normalized groups
	Ctrl	31781.66	1.091680203	1	38884.635	0.989379206	1	29330.539	0.82922317	1	1	0
	PAE	20474.51	0.745617295	0.68299974	27725.522	0.677346744	0.684617929	19286.246	0.592991256	0.715116602	0.694244757	0.018093647
	Nilotinib	20648.359	0.717221684	0.656988816	30605.456	0.744825348	0.752820903	24332.782	0.744929601	0.898346341	0.769385353	0.12152839
CR-ABL(Input)												
	Ctrl	29112.61	0.916019176	1	39302.054	1.010734806	1	35371.104	1.205947971	1	1	0
	PAE	27459.811	1.341170607	1.464129401	40932.539	1.476348723	1.460668728	32523.66	1.686365506	1.398373352	1.44105716	0.037005739
	Nilotinib	28789.368	1.394269055	1.522095926	41090.782	1.342596627	1.328337186	32664.539	1.342408731	1.113156424	1.321196512	0.204563245
ıultiple comı	Mean Diff.	95.00% CI of diff.	Significant?	Summary	djusted P Valı	A-?						
CTRL vs. PAI	0.3035	0.09185 to 0.5151	Yes	*	0.0113	B	PAE					
RL vs. Niloti	0.2281	0.01650 to 0.4397	Yes	*	0.0377	C	Nilotinib					
Test details	Mean 1	Mean 2	Mean Diff.	SE of diff.	n1	n2	q	DF				
CTRL vs. PAI	1	0.6965	0.3035	0.07391	3	3	4.106	6				
RL vs. Niloti	1	0.7719	0.2281	0.07391	3	3	3.086	6				
Ctrl	1	1	1	1	0							
PAE	0.6829997	0.684617929	0.715116602	0.694244757	0.018093647	*						
Nilotinib	0.6569888	0.752820903	0.898346341	0.769385353	0.12152839	*						

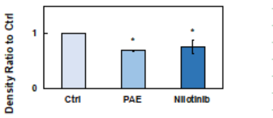

Supplement Figure S6 Original unedited bands for evaluating PAE treatment affected related signaling pathways proteins measured by western blotting in Fig. 6C. Image software was used for sensitivity analysis, and the relative quantitative statistics and normalized the intensity value against “Ctrl” group were adopted. Western blotting was performed 3 times. The “Repeat 1” was used in the manuscript.
